# Enhanced TrkA signaling impairs basal forebrain-dependent behavior

**DOI:** 10.3389/fnmol.2023.1266983

**Published:** 2023-09-22

**Authors:** Laura Calvo-Enrique, Silvia Lisa, Cristina Vicente-García, Ruben Deogracias, Juan Carlos Arévalo

**Affiliations:** ^1^Department of Cell Biology and Pathology, Instituto de Neurociencias de Castilla y León (INCyL), Universidad de Salamanca, Salamanca, Spain; ^2^Institute of Biomedical Research of Salamanca (IBSAL), Salamanca, Spain

**Keywords:** TrkA, NGF (nerve growth factor), basal forebrain, cholinergic neurons, mice behavior, learning

## Abstract

Basal forebrain cholinergic neurons (BFCNs) modulate cognitive functions such as attention, learning and memory. The NGF/TrkA pathway plays an important role in the development and function of BFCNs, although two mouse models conditionally deleting TrkA expression in the central nervous system (CNS) have shown contradictory results. To shed light into this discrepancy, we used a mouse model with a gain-of-function in TrkA receptor signaling. Our results indicate that enhanced TrkA signaling did not alter hippocampal cholinergic innervation, general locomotion or anxiety-related behaviors, but it increases ChAT expression, the number of cholinergic neurons at early postnatal stages and, mutant mice showed impaired motor learning and memory functions. These data demonstrate that proper functioning of the cholinergic system in CNS requires a balanced NGF/TrkA signaling.

## Introduction

Nerve growth factor (NGF) exerts its functions through two different receptors: the tropomyosin-receptor kinase, TrkA, and the pan-neurotrophin receptor p75^NTR^ (Arevalo and Wu, 2006; Reichardt, 2006). The *in vivo* function of NGF through TrkA is well understood in the peripheral nervous system (PNS), acting as a survival factor for sensory and sympathetic neurons (Crowley et al., 1994; Smeyne et al., 1994). However, in the central nervous system (CNS), the role of NGF/TrkA signaling is more elusive and sometimes involved in controversy. TrkA expression in the CNS is restricted mainly to cholinergic neurons in the basal forebrain (BF) and striatum (Sobreviela et al., 1994). The BF is the principal source of cholinergic innervation in the CNS playing a critical role in learning, memory, and attention (Linville and Arnerić, 1991; Baxter and Chiba, 1999; Ballinger et al., 2016). The basal forebrain cholinergic neurons (BFCNs) project mainly to the hippocampus and throughout the cortex (Ballinger et al., 2016), from which they receive NGF that is retrogradely transported back to the cell bodies (Seiler and Schwab, 1984; DiStefano et al., 1992; Sobreviela et al., 1996). The role of this NGF/TrkA complex in BFCNs has been controversial in Alzheimer’s disease (AD) (Abubakar et al., 2022). Importantly, axonal transport through the septo-hippocampal tract is impaired and reduced *in vivo* with normal aging in mice and is aggravated by AD pathology (Bearer et al., 2018).

The role of NGF/TrkA signaling in the BFCNs has been addressed using mainly the TrkA and NGF knockout mice (KO). There is a reduction on neuronal survival and neuronal atrophy (Fagan et al., 1997), reduced levels of ChAT (choline-acetyl transferase) expression (Crowley et al., 1994; Smeyne et al., 1994) and a reduction in the projections to the hippocampus and cortex at P20–P25 (Fagan et al., 1997). However, these studies have limitations since KO mice only survive for a month. To overcome this problem, conditional KO mice in the CNS have been used. Sanchez-Ortiz and colleagues utilized the Dlx5/6-Cre mouse to remove TrkA expression from E16.5 (Sanchez-Ortiz et al., 2012), whereas Müller and colleagues utilized a Nestin-Cre to delete TrkA or NGF from E11.5 (Müller et al., 2012). Both studies agreed that ChAT expression and hippocampal and cortical innervation are reduced. However, Müller and colleagues found a decrease in the number of cholinergic neurons whereas Sanchez-Ortiz and colleagues did not. These two studies differ in the results mostly from mouse behavior experiments. Sanchez-Ortiz and colleagues observed differences between control and mutant mice in the tone-cue fear-conditioning test and in the novel object recognition (NOR) task (Sanchez-Ortiz et al., 2012), whereas Müller and colleagues, did not find differences in any of the behavioral tests assessed (Müller et al., 2012). In addition, Yanpallewar and colleagues have used a gain-of-function TrkA mouse model, which has a deletion of three amino acids in the juxtamembrane region of TrkA leading to an increase in the receptor levels and in ChAT expression (Yanpallewar et al., 2022). In the behavioral experiments, they found no alterations in locomotor activity nor in anxiety-nor in depression-related behaviors, but they showed that mutant mice have attenuated fear responses in the fear-conditioning test (Yanpallewar et al., 2022). Therefore, there are conflicting results between these studies regarding the role of NGF/TrkA signaling in the CNS.

To clarify these discrepancies, we used a gain-of-function mouse model for TrkA signaling, TrkAP782S (KI), which has an impact on TrkA activation, trafficking, and nociceptive behavior (Yu et al., 2011, 2014). We monitored the number of TrkA-, ChAT- and p75^NTR^-expressing neurons, TrkA and ChAT protein levels, as well as cholinergic hippocampal innervation in KI mice and control littermates at different ages. In addition, we have assessed general and learning locomotion, anxiety, and novel object recognition (NOR) task. We have observed that ChAT expression is increased at P15 in KI mice and that motor learning and cognition in NOR tests are impaired in mutant mice. Our data confirmed the role of NGF/TrkA signaling in early ChAT expression and suggest that proper, controlled NGF/TrkA signaling in CNS is important for learning and memory functions.

## Materials and methods

### Mice

All the experimental procedures were performed following the European Community guidelines (63/2010), the Spanish Royal Decree 53/2013 and the Order ECC/566/2015. Protocols were previously approved by the Bioethics Committee of the University of Salamanca. Mice, with a maximum of 5 animals per cage, were housed in SPF Animal Facility of the University of Salamanca. They were fed *ad libitum* in a 12 h-light/dark cycle, kept at constant temperature of 20–22°C and a relative humidity of 55–65%. Only male mice were used throughout the study. The *TrkA-P782S* (KI) mice were previously described (Yu et al., 2011, 2014) and wild type littermates were used as controls. *TrkA-Cre* mice (*B6; 129S4-Ntrk1^tm1(cre)Lfr^/Mmucd*, MMRRC #015500-UCD) were crossed with *RCL-tdT* mice (B6.Cg-*Gt (ROSA)26Sor^tm9(CAG-tdTomato)Hze^*/J, JAX007909) (Madisen et al., 2010) to generate *TrkA-Cre; RCL-tdT* mice. In these mice, we identified neurons that have expressed or actually express TrkA.

### Histology

Mice were transcardially perfused with cold 0.1 M phosphate buffer (PB) pH 7.4, followed by 4% paraformaldehyde (PFA) in 0.1 M PB. Brains were extracted, post-fixed overnight in 4% PFA in 0.1 M PB at 4°C and then incubated in PBS with 30% (w/v) sucrose at 4°C until they sank. Each brain was coronally sectioned at 40 μm and floating sections were stored in cold 0.1 M PB containing 0.02% NaN_3_.

For 3,3′-diaminobenzidine-based immunostaining, sections were incubated in 0.3% hydrogen peroxide for 10 min at room temperature (RT) to reduce activity of endogenous peroxidase, then washed with PBS with 0.2% Triton X-100 and blocked in PBS with 5% normal goat serum (NGS), 1% bovine serum albumin (BSA) and 0.2% Triton X-100 for at least 2 h at RT. Antibody incubations were performed in PBS containing 2% NGS, 1% BSA and 0.2% Triton X-100 at 4°C with gentle agitation for 48–72 h. The following antibodies were used: rabbit ChAT (1,500, Ab143, Millipore), rabbit TrkA (1:500; gift from L.F.Reichardt, University of California, San Francisco, CA; Clary et al., 1994), rabbit p75^NTR^ (9992; 1:500; gift from M.V.Chao, Skirball Institute, NYU Medical Center, NY; Benedetti et al., 1993). Biotin-conjugated secondary antibodies (1:500, Cat. 111-065-003, The Jackson Laboratory) were incubated at RT for 1 h 30 min. The staining was developed using Vectastain ABC system (Vector Laboratories) and sections were mounted on gelatin-coated slides, dehydrated and cover-slipped with Entellan (Merck).

For immunofluorescences (IF), floating sections were incubated with primary antibodies as described above overnight at 4°C and Alexa-labeled secondary antibodies (1:750, Cat. A11008 and A 21424, Invitrogen) were utilized for 1 h 30 min at RT, counterstained with Hoechst 33342 (1:13000) and mounted on gelatin-coated slides with Vectashield (Vector Laboratories). The following antibodies were used for IF: rabbit TrkA (1:500; gift from L.F.Reichardt, University of California, San Francisco, CA; Clary et al., 1994) and Nedd4-2 (15 μg/mL; Arevalo et al., 2006). Images were collected using an Olympus AX70 microscope equipped with an Olympus DP70 camera for inmunohistochemistry stainings or with a Leica STELLARIS DMI8 confocal microscope for inmunofluorescences.

### Quantification of cholinergic neurons from the medial septum & diagonal band nucleus

To quantify cholinergic neurons in MS and DB, coronal brain sections from WT and TrkAP782S mice spanning from bregma 0.20 to 1.50 mm were immunostained for ChAT, TrkA or p75^NTR^. Positive cells for each staining were counted in the whole MS and vertical and horizontal limb of the DB. These regions were defined according to the mouse brain atlas (Paxinos and Franklin, 2001). A section every 280 μm was counted, for a total of 3–4 sections throughout the rostrocaudal extent of the MS and the DB. The cell counting was carried out using ImageJ software by two independent investigators blind to the genotyping and the results presented are the average of both researchers.

### Quantification of cholinergic nerve terminal density in dorsal hippocampus

The density of cholinergic nerve terminals in the *stratum oriens* of the CA1 region of the hippocampus was evaluated from sections stained for p75^NTR^ spanning approximately from bregma-1,2 to-2.5 mm. Nerve terminal density was obtained using the software Photoshop measuring the integrated density of p75^NTR^ staining above threshold.

### Preparation of protein lysates

Mice were killed by cervical dislocation and brains were quickly dissected. Specific areas of the brain were snap-frozen in liquid nitrogen and homogenized in lysis buffer containing 10 mM Tris pH 7.4, 150 mM NaCl, 2 mM EDTA, 1% NP-40, 0.1% SDS, 1 mM PMSF, 1 μg/mL aprotinin, 2 μg/mL leupeptine, 1 mM vanadate, 10 mM NaF, and 20 mM β-glycerophosphate at 4°C and passed through a 26G needle to dissociate. Lysates were centrifuged at 14,000 rpm for 15 min at 4°C to eliminate the debris.

### Activation assay

MS from P15 WT and KI littermates were collected in 0.9% sodium chloride (Vitulia, Cat. #999791.5). After 20 min in sodium chloride solution at RT, we performed NGF treatment with mouse NGF 2.5S (Cat. N-100; Alomone labs) at 25 ng/mL or 100 ng/mL for 15 min at RT with gentle agitation. Finally, NGF was removed and tissue was properly washed. Protein lysates preparation was performed as described above and WB were performed.

### Western blot analysis

Protein concentration was determined using the Bradford protein assay (Bio-Rad). Laemmli buffer was added to each sample, boiled for 5 min to denature proteins that were resolved by SDS-PAGE and immunoblotted with specific antibodies. The following antibodies were used: rabbit p42/44 MAPK (ERK1/2) (1:1000, #9102, Cell Signaling Technology), mouse phospho-p42/44 MAPK (ERK1/2) (1:1000, #9106, Cell Signaling Technology), ChAT (1,1,000, Ab144, Millipore), β-actin and β-tubulin III (1:5000 and 1:10000, #A-4700, #T2200 respectively, Sigma-Aldrich), TrkA (RTA; 1:500; gift from L. Reichardt USCF, San Francisco, CA; Clary et al., 1994), rabbit Nedd4-2 (1: 3,000; Arevalo et al., 2006) and rabbit p75^NTR^ (9992; 1:1000; gift from M.V.Chao, Skirball Institute, NYU Medical Center, NY; Benedetti et al., 1993). Horseradish peroxidase-conjugated secondary antibodies were used at a dilution 1:10000. Blots were then processed by the ECL chemiluminescence method and the intensity bands were quantified as reported (Cañada-García and Arévalo, 2023).

### Behavioral assays

All tests were conducted during the light cycle by an experimenter blind to the genotype. Mice were habituated to handling for at least 2 days before behavioral tests began. Mice were given 1 h to habituate to the behavioral room before any test was conducted. Behaviors were videotaped and analyzed using ANY-Maze software.

### Open field test

The open field test was performed in an arena made of plexiglas (50 × 40 cm) surrounded by walls (30 cm high). Animals were placed next to a corner and allowed to freely explore for 10 min. The center area of the chamber was designated as 30 × 20 cm. The total distance covered and the time spent at the center was quantified.

### Elevated plus maze test

The elevated plus maze test was performed in a dark plexiglas elevated maze with four arms (30 cm long and 5 cm wide) extending from a central platform (5 × 5 cm). While two facing arms were closed by dark walls (15 cm high), the other two arms were open. Animals were placed in the center of the apparatus and allowed to explore it for 10 min. The time spent in open and closed arms was analyzed.

### Novel object recognition test

Mice were allowed to explore the open field arena described above for 10 min. Next day, during the sample phase, mice were exposed to a pair of identical objects placed in the back left and right corners of the arena for 5 min. Object recognition memory (ORM) was tested 1 h after this trial by exposure to a previously familiar object and to a new one for 5 min. Familiar and novel objects were counterbalanced. The snouts of the mice were tracked, and object interactions were measured as time spent with snout within 2 cm of the object. The discrimination ratio was b/(a + b) where a is the exploration time of the familiar object and b is the exploration time of the novel object. ORM was measured as the increased time spent investigating the novel object.

### Rotarod test

Motor coordination task was performed using an accelerating rotarod. The test assesses fore-and hind-limb balance coordination as well as a learning component for this motor task (Deacon, 2013). The rotation rate of the cylinder increased from 4 to 40 rpm over a 5 min period. The latency of each mouse to fall was automatically recorded by the device. The test was performed for four trials a day for 4 consecutive days.

### Statistical analyses

For neuronal counts and biochemical analysis, experiments were performed on a minimum of three animals for each genotype and age. Data are presented as mean ± SEM, unless otherwise mentioned in the figure legend. Statistical significance was analyzed using GraphPad Prism 6 (GraphPad software). Comparisons between means were assessed by different statistical test described at the figure legend after having checked normality of data. For those data with small numbers or non-normal distribution, comparisons were done with a non-parametric test.

## Results

### Increased activation of TrkAP782S in the medial septum

**Figure 1:**
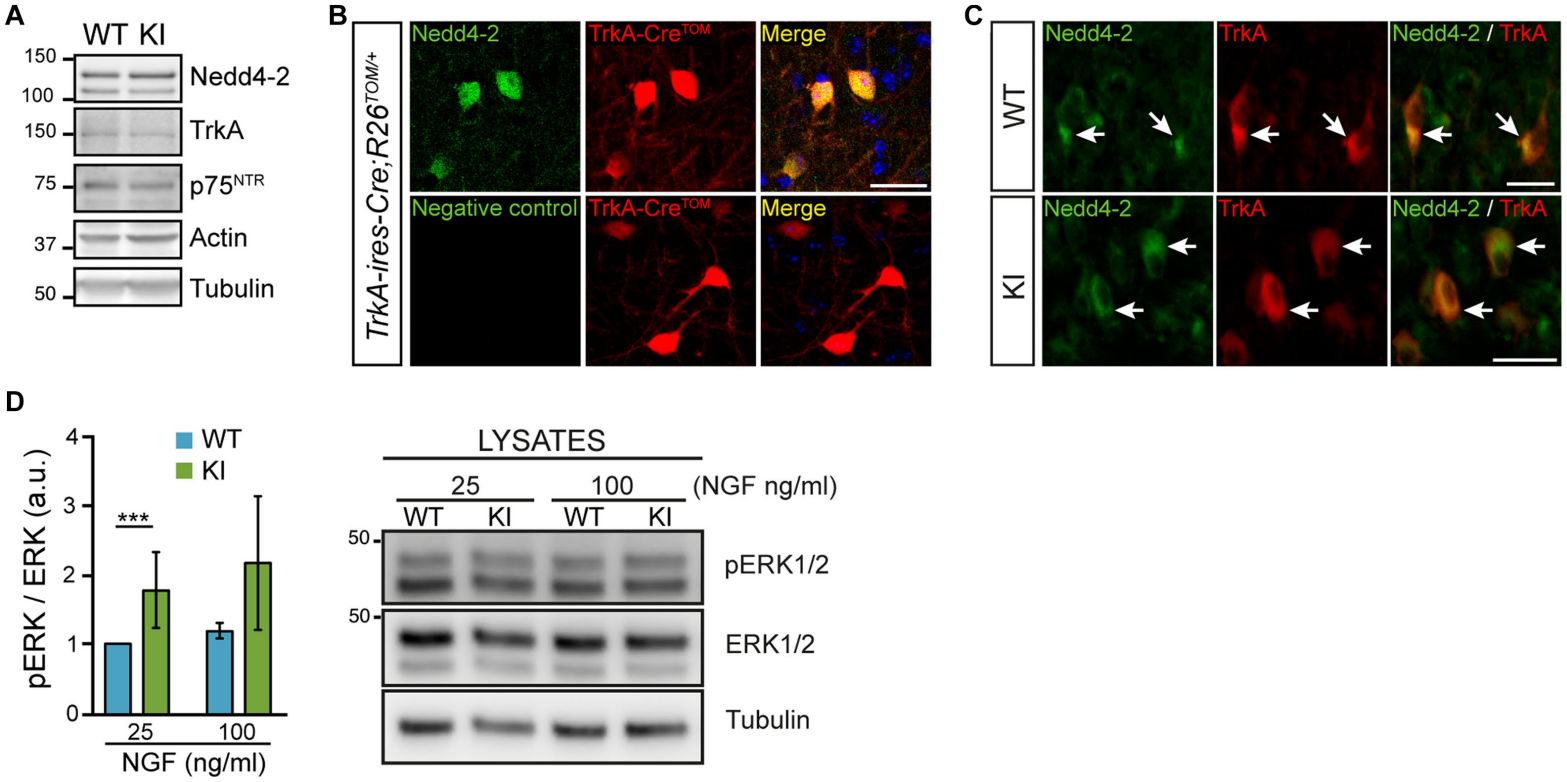
Nedd4-2 expression and TrkAP782S receptor activation in the cholinergic neurons of the basal forebrain. **(A)** TrkA, p75^NTR^ and Nedd4-2 are expressed in the basal forebrain of 2 months-old TrkA WT and KI mice. A representative Western blot is shown (*n* = 4, for each genotype). Actin and tubulin were used as loading controls. **(B)** Nedd4-2 is expressed in TrkA-positive neurons which are labeled with tdTomato. Immunofluorescence against Nedd4-2 (upper panels) and without primary antibody (negative control, lower panels) in 40 μm brain slices from *TrkA-ires-Cre; R26Tomato^+/−^* mice. Scale bar: 50 μm. **(C)** Nedd4-2 is co-expressed with TrkA in neurons from medial septum region of WT and KI mice. Immunofluorescence against Nedd4-2 and TrkA in brain slices from WT and KI mice. White arrows point to double stained neurons. Scale bar: 25 μm. **(D)** TrkAP782S protein is more active than WT in the basal forebrain of mice. Basal forebrain sections from WT and KI mice were stimulated for 15 min with NGF (25 and 100 ng/mL) and lysates were obtained. Quantification of ERK1/2 activation (pERK/ERK at protein levels) is shown (*n* = 4, non-parametric Wilcoxon signed rank test, *p* < 0.0001). A representative Western blot is shown for ERK1/2 activation in WT and KI mice.

**Figure 2:**
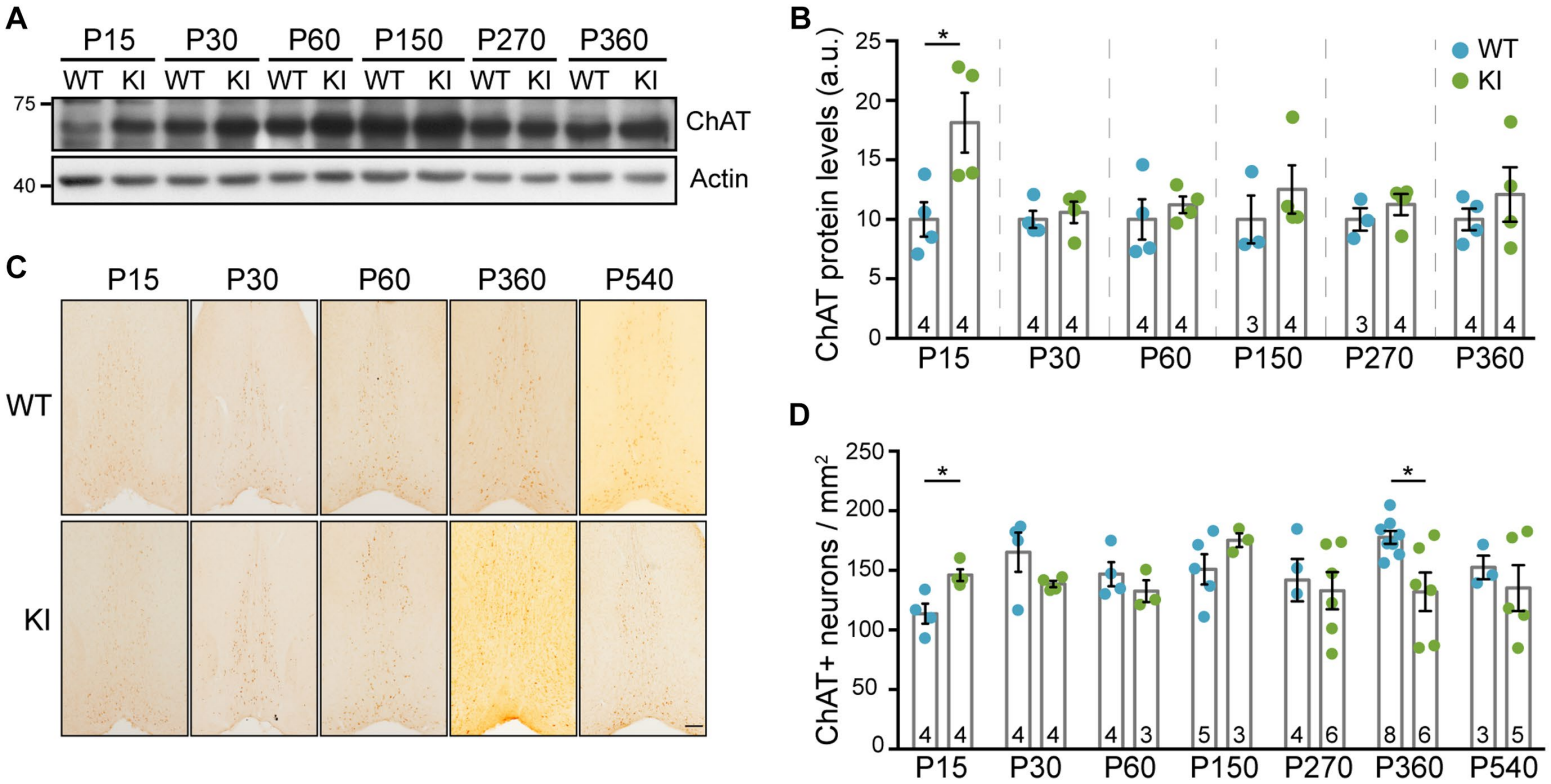
ChAT protein levels and the number of cholinergic neurons are increased in TrkA KI mice at P15. **(A)** Western blot showing ChAT protein levels at different ages in WT and KI mice. Actin was used as loading control. **(B)** Quantification of ChAT protein levels (*n* = 3–4 per age and genotype as indicated in inside bars). Two-way ANOVA *F* (1,34) = 8.441 and *p* = 0.0064 (**) for genotype. Turkey’s multiple comparisons test WT vs. KI at P15 *p* < 0.05. Protein levels from KI mice are compared against WT littermates at every age. **(C)** Representative images of immunohistochemistry against ChAT in the MS of WT and KI mice at different ages. **(D)** Quantification of ChAT-positive neurons per area (*n* = 3–8 per age and genotype as indicated in inside bars). Mann–Whitney U test, *p* = 0.0296 (WT vs. KI at P15) and *p* = 0.020 (WT vs. KI at P360).

Upon NGF binding, TrkA is ubiquitinated by different E3 ubiquitin ligases (Geetha et al., 2005; Makkerh et al., 2005; Arevalo et al., 2006). One of those E3 ubiquitin ligases is Nedd4-2. Nedd4-2 binds to the TrkA PPXY motif and modification of this motif prevents Nedd4-2 binding and renders a gain-of-function TrkA receptor as in the case of TrkAP782S (Arevalo et al., 2006). To study *in vivo* the NGF/TrkA signaling, we generated a mutant mouse (TrkAP782S, hereinafter TrkA KI) unable to bind Nedd4-2, yielding an overactivated TrkA receptor in the PNS (Yu et al., 2011, 2014). To assess whether increased NGF/TrkA signaling has any effect on CNS, we performed different experiments. But first, we checked Nedd4-2 expression in the basal forebrain, particularly in the MS region where TrkA is expressed, with several approaches: (1) using lysates from the basal forebrain of 2 months old mice, we detected Nedd4-2 expression as well as TrkA and p75^NTR^ in both WT and TrkA KI samples (Figure 1A); (2) we stained with Nedd4-2 antibodies brain slices from the *TrkA-Cre;RCL-tdT* reporter mice where TrkA-positive cells are labeled with the fluorescent protein Tomato (Figure 1B); and (3) we co-stained with TrkA and Nedd4-2 antibodies in brain slices from control and TrkA KI mice (Figure 1C). The results indicated that Nedd4-2 is co-expressed with TrkA-positive neurons in the MS. To address whether TrkAP782S receptor is more active than the WT in the MS, brain slices were stimulated with different amounts of NGF for 15 min and lysates were obtained. We observed that activation of ERK1/2, which is downstream of NGF/TrkA signaling, was increased in TrkA KI samples (Figure 1D). Therefore, TrkAP782S protein is more active than the WT in cholinergic neurons from the MS. Altogether these data indicate that Nedd4-2 modulates TrkA activation in MS.

### The expression of TrkAP782S in basal forebrain increases ChAT levels and the number of cholinergic neurons

To assess whether enhanced NGF/TrkA signaling has any effect on cholinergic neurons, we used different approaches using TrkA KI mice and WT littermates as controls. First, we assessed ChAT protein levels in lysates from MS at different ages (Figure 2A) and detected a significant increase exclusively at P15 in mutant mice (Figure 2B). To address whether this enhancement was due to a difference in the number of ChAT-positive neurons, we stained brain sections from WT and TrkA KI mice (Figure 2C). We found that in TrkA KI mice ChAT-positive neuron number was significantly increased at P15 (Figure 2D), which correlates with the increase amount of ChAT at this age. However, at P360 there is a reduced number of ChAT-positive neurons in TrkA KI mice that it is not maintained at P540. Since cholinergic neurons in the MS express TrkA (Sobreviela et al., 1994), we assessed TrkA protein levels and also performed immunohistochemistry using WT and TrkA KI mice from P15 up to P540. We did not observe differences at the protein level (Supplementary Figures S1A,B) nor at the number of TrkA-positive neurons (Supplementary Figures S1C,D). Therefore, TrkA-enhanced signaling increases ChAT protein levels and the number of cholinergic neurons during early postnatal development.

**Figure 3:**
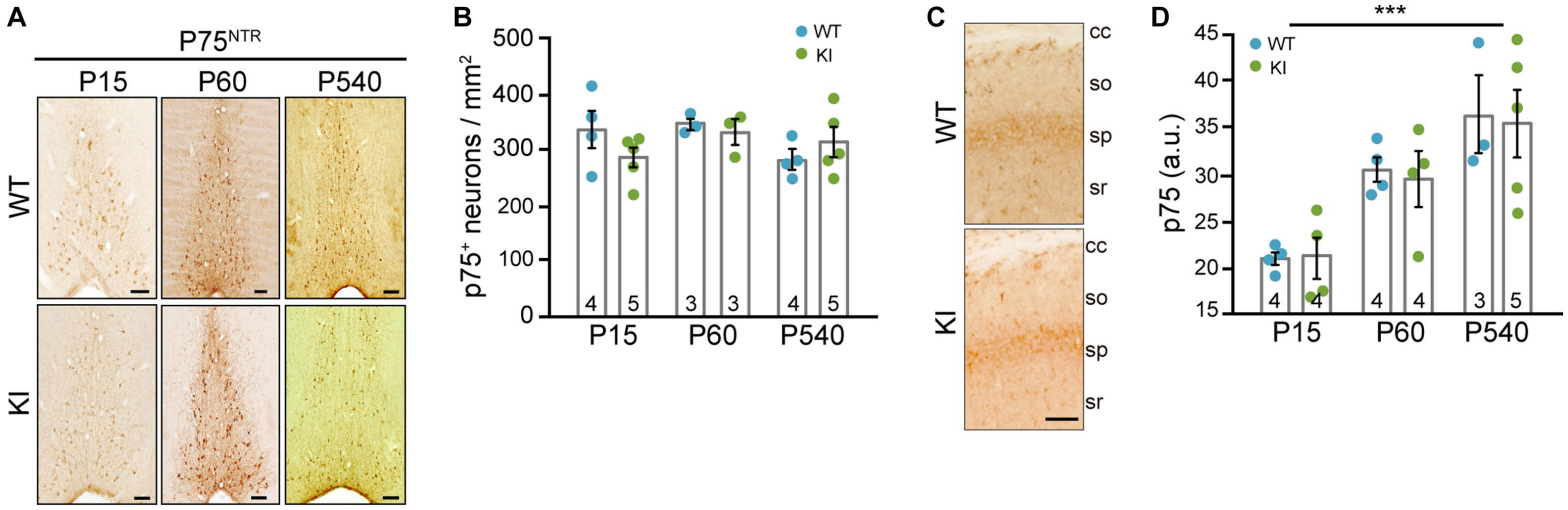
p75^NTR^-expressing neurons in MS and cholinergic innervation of hippocampal CA1 region in TrkA KI mouse are not altered. **(A)** Representative images of immunohistochemistry against p75^NTR^ in the MS of WT and KI mice at different ages. **(B)** Quantification of p75^NTR^-positive neurons per area at different ages in WT and KI mice (*n* = 3–5 per age and genotype as indicated in inside bars). **(C)** Representative image of immunohistochemistry against p75^NTR^ in the CA1 region of KI mice. Scale bar: 50 μm. **(D)** Quantification of CA1 innervation by p75^NTR^-positive fibers per area in WT and KI mice (*n* = 3–5 per age and genotype as indicated in inside bars). Two-way ANOVA *F* (2, 18) = 14.42 and *p* = 0.0002 for age.

It is known that cholinergic neurons from the MS project into the dorsal hippocampus, into CA1 region (Amaral and Kurz, 1985; Yoshida and Oka, 1995; Müller and Remy, 2018), so we decided to test these projections. Knowing that p75^NTR^ is expressed in these cholinergic neurons of the MS (Sobreviela et al., 1994), we stained sections with an antibody against p75^NTR^. First of all, we quantified the number of p75^NTR^-positive neurons in the MS of TrkA WT vs. KI mice and we did not find differences between genotypes at any ages (Figures 3A,B). Then, we quantified the projections into the CA1 region of the hippocampus and found no differences between WT and KI mice (Figures 3C,D). Thus, a mutant TrkA leading to overactivation in cholinergic neurons does not alter the number of p75^NTR^-positive neurons nor innervation of CA1 region.

### TrkAP782S expression does not alter general locomotion nor anxiety

**Figure 4:**
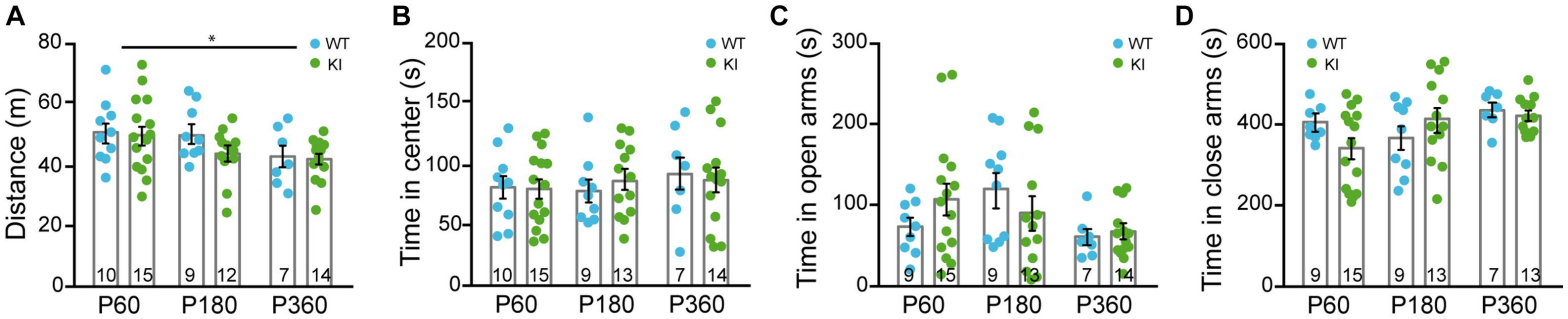
General locomotion and anxiety-related behaviors are similar in TrkA WT and KI mice. **(A,B)** Open field assay performed with TrkA WT and KI mice at P60, P180, and P360 in which: **(A)** total distance traveled, Two-way ANOVA *F* (2, 60) = 3.940 an *p* = 0.0247 for age and **(B)** time spend in the central area were assessed. **(C,D)** Elevated plus maze assay showing time spent in **(C)** open arms and **(D)** closed arms by WT and KI mice (*n* = 9–15, as indicated in inside bars).

To assess general locomotion, we performed the open field test in TrkA WT and KI mice. Mice from both genotypes covered similar total distance at P60, P180, and P360 (Figure 4A), with a slight decrease due to aging in both genotypes, and spent the same amount of time in the center of the arena (Figure 4B). We also carried out experiments using the elevated plus maze to test their anxiety-like behavior and we did not observe differences in the time spent in the open or closed arms between genotypes at the aforementioned ages (Figures 4C,D). Therefore, TrkAP782S mice do not show alterations in general locomotion nor anxiety.

### TrkAP782S expression impairs motor learning

**Figure 5:**
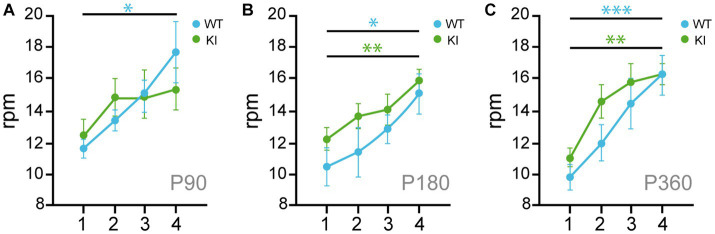
Transient locomotor learning impairment in KI mice at P90. **(A–C)** Rotarod assays performed in 4 consecutive days with TrkA WT and KI mice at P90 **(A)**, P180 **(B)**, and P360 **(C)** (*n* = 7–14). Unpaired Student’s *t*-test with Welch’s corrections * *p* < 0.05; ** *p* < 0.01; *** *p* < 0.001.

To assess motor skill coordination, we performed rotarod accelerated task. TrkA WT and KI mice show similar locomotor coordination at P90, P180, and P360 (Figures 5A–C), but the mutant mice at P90 did not improve their motor coordination after 4 sessions of training (4 trials per session) as the TrkA WT did (Figure 5A). Therefore, TrkA KI mice show a transient deficit in locomotor learning at P90.

### Impaired attention and memory in TrkA KI mice

**Figure 6:**
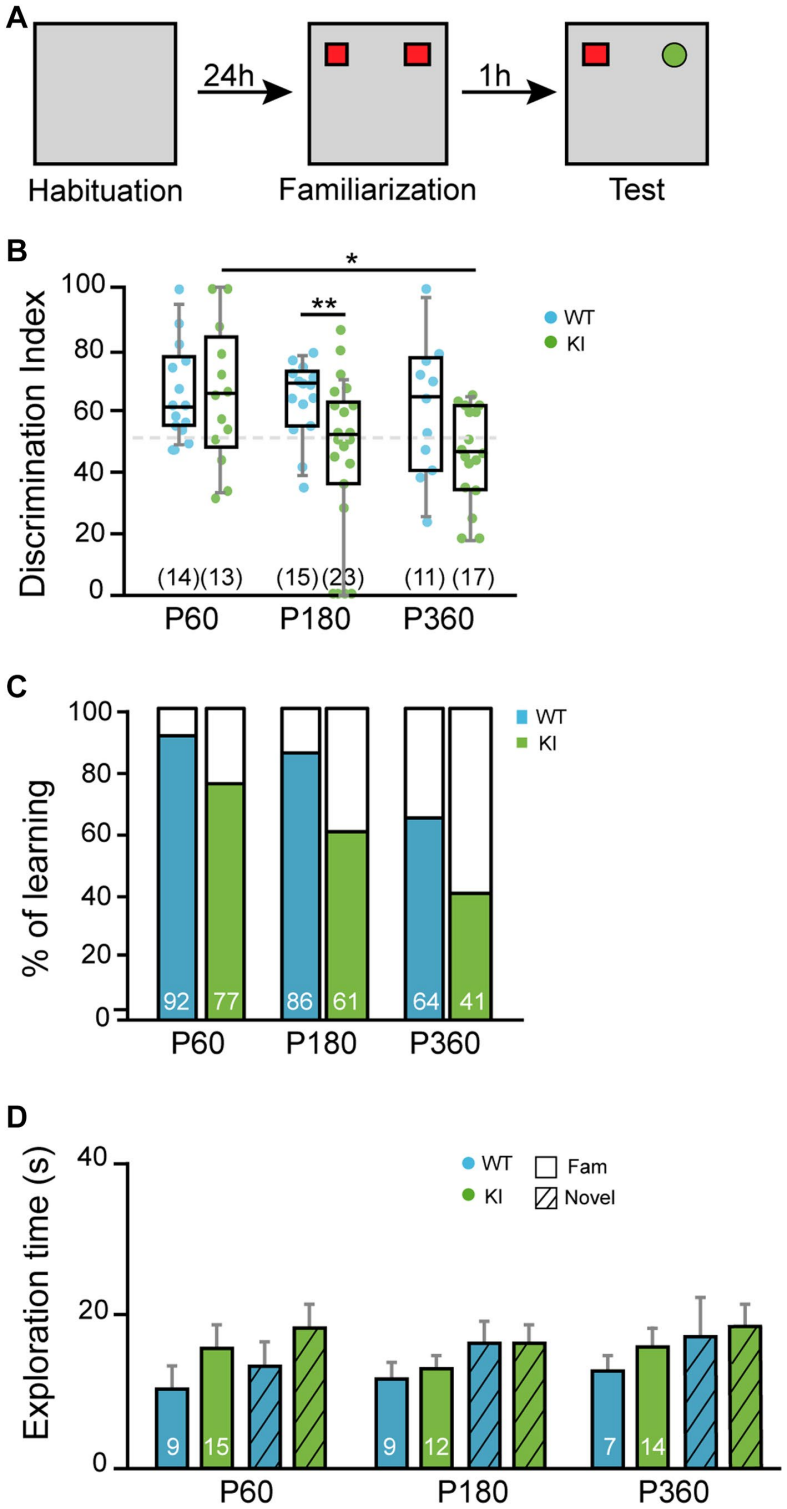
Novel object recognition is deficient in KI mice. **(A)** Scheme of novel object assay. **(B)** Discrimination index obtained with TrkA WT and KI mice at P60, P180, and P360 (*n* = 13–23). Unpaired Student’s *t*-test with Welch’s correction, *p* = 0.005WT vs. KI at P180, *p* = 0.0157 P60 vs. P360 in KI. Data are presented in a box and whiskers plot with 10-90% percentil. **(C)** Percentage of mice that spent more time exploring the novel object than the familiar one. **(D)** Total time of exploration. Total number of mice per condition are indicated inside brackets. Note that TrkA KI mice at P180 did not perform as well as TrkA WT animals while they expend more time exploring the objects.

Cholinergic neurons from MS have been implicated in different behaviors such as attention and memory (Ananth et al., 2023). To assess the increase of NGF/TrkA signaling in MS in the working memory and the attention system, we performed the NOR test (Figure 6A). We measured the discrimination ratio between the novel and familiar object. At P60, TrkA WT and KI mice performed similarly, whereas at P180 the KI ones showed a significant deficit in recognizing the novel object (*p* = 0.005, KI vs. WT mice) and a tendency at P360 (*p* = 0.0736, KI vs. WT mice) (Figure 6B). In addition, the percentage of TrkA KI mice that discriminate the novel object vs. the familiar one above 50% was reduced at every age compared. At P60 (77% vs. 92%, KI vs. WT), at P180 (61% vs. 86%, KI vs. WT mice) and at P360 (41% vs. 64%, KI vs. WT mice) (Figure 6C). These deficits were not due to lack of exploration since KI mice explored as much as WT, both novel and familiar objects, in every experimental age (Figure 6D). Therefore, an enhanced NGF/TrkA signaling in cholinergic neurons leads to an impairment in memory and attention in mice.

## Discussion

Several transgenic mouse models have been used along the literature to address the role of TrkA expression and activation in the BFCNs (Crowley et al., 1994; Smeyne et al., 1994; Fagan et al., 1997; Müller et al., 2012; Sanchez-Ortiz et al., 2012; Yanpallewar et al., 2022). The results obtained from them can be summarized as: (1) TrkA modulates ChAT expression during early postnatal development, (2) the size of the cells from mutant mice is smaller in KO mice, and (3) cholinergic hippocampal innervation is reduced in KO mice. However, there are discrepancies regarding the role of TrkA and NGF in the BFCN survival and in the behavior of mice lacking TrkA or NGF. Most of these studies have used a loss-of-function approach to tackle these questions, whereas in the present article, we have utilized a mutant TrkA mouse with a gain-of-function previously reported in the PNS (Yu et al., 2011, 2014) and now in the CNS (Figure 2). Therefore, we have a mouse model with a TrkA overactive receptor for the *in vivo* study of TrkA role in CNS.

Loss of TrkA or NGF causes a marked decrease in ChAT levels in BFCNs when TrkA is deleted (Crowley et al., 1994; Smeyne et al., 1994; Müller et al., 2012; Sanchez-Ortiz et al., 2012) and an increase when TrkA is overactive (Yanpallewar et al., 2022), indicating a critical role of NGF/TrkA signaling in the expression of ChAT. This is supported by our study using a gain-of-function of TrkA, which leads to an increase in ChAT levels in early postnatal stages (Figures 2A,B). Altogether these data indicate that ChAT expression in BFCNs unequivocally depends on NGF/TrkA signaling. In addition, our results indicate a transient increase in the number of BFCNs at P15 (Figures 2C,D). It has been reported that TrkA signaling can compete with p75^NTR^ signaling preventing apoptosis (Deppmann et al., 2008; Singh et al., 2008). Deletion of p75^NTR^ in ChAT-positive neurons led to a significant increase in neuron number (Boskovic et al., 2014), demonstrating a role of p75^NTR^ in promoting the programmed cell death of cholinergic neurons that occurs at early postnatal stages (Ward and Hagg, 1999). Therefore, our TrkA mouse model seems to counteract the deleterious effect of p75^NTR^. Additional experiments will be required to further confirm this hypothesis. Our results also agree with those ones obtained using the full TrkA KO (Fagan et al., 1997) or the conditional deletion of TrkA from Nestin-expressing cells (Müller et al., 2012), which reported 36% fewer ChAT-IR neurons in the septum of TrkA KO mice compared with WT. However, Sanchez-Ortiz and colleagues did not observe differences in the number of cholinergic neurons (Sanchez-Ortiz et al., 2012). This may be due to the late deletion of TrkA carried out under Dlx5/6 promoter.

BFCNs from MS project to different areas of the brain including the hippocampus (Ballinger et al., 2016). Our results suggest that overactivation of TrkA does not affect BFCNs target projection to the hippocampus (Figure 3). However, TrkA deletion led to an impairment in cholinergic projections to the hippocampus and prefrontal cortex (Smeyne et al., 1994; Müller et al., 2012; Sanchez-Ortiz et al., 2012). Therefore, we can speculate that the increased TrkA signaling does not elicit an enhanced response regarding cholinergic innervation.

BFCNs are involved in learning, cognition, and memory (Ananth et al., 2023). Therefore, we tested the KI mouse behavioral performance observing no defects on general locomotion, anxiety-related behaviors, and motor coordination (Figures 4, 5), similar to what Yanpallewar and colleagues reported. However, in our study we observed impairments in motor learning (Figure 5A), and NOR task (Figure 6). Whereas Sanchez-Ortiz and colleagues, found that TrkA absence in cholinergic neurons resulted in a significant decrease in the recognition of novelty compared with control mice (Sanchez-Ortiz et al., 2012), Müller and colleagues observed that TrkA signaling is unnecessary for attention behavior and different aspects of learning and memory in adult animals (Müller et al., 2012). In addition, Yanpallewar and colleagues did not find any defect on the NOR task in mice at P90 (Yanpallewar et al., 2022), whereas we detected deficits at P180 and P360, but not at P60 (Figures 6B,C). Altogether these studies indicate that both gain-and loss-of function in TrkA signaling leads to deficits in behavior suggesting that proper activation levels of TrkA are required for correct behavior.

Our results indicate a transient increase in ChAT expression at P15 (Figure 2) in KI mice whereas the behavioral experiments performed from P60 showed several deficits in the KI mice (Figures 5, 6). To explain and compare these different set of data, we postulate that the impaired behavior may be due not the early enhanced expression of ChAT elicited by enhanced NGF/TrkA signaling but to its regulatory effect on acetylcholine release as previously reported (Auld et al., 2001a,b). Therefore, enhanced NGF/TrkA-mediated signaling may result in different outcomes at different ages.

Several studies have suggested that NGF and its receptors, TrkA and p75^NTR^, may be involved in the pathogenesis of Alzheimer’s disease (AD) (Mufson et al., 2008; Cattaneo and Calissano, 2012). Whereas NGF/TrkA signaling could function in a neuroprotective role during aging and/or in neurodegenerative diseases (Mufson et al., 1999; Sofroniew et al., 2001), p75^NTR^ could promote apoptosis (Wong et al., 2022). BF dysfunction seems to be predictive of AD as demonstrated by different studies (Teipel et al., 2014; Baker-Nigh et al., 2015; Ballinger et al., 2016; Schmitz et al., 2016). NGF receptor variations have been observed before phenotypic changes of BFCNs in AD, suggesting that a lack of neurotrophic support is instrumental with respect to basal forebrain degeneration (Mufson et al., 2000, 2003). Therefore, disbalance in neurotrophins and their receptors may play a role in AD. Aside from AD, age itself has been shown to negatively impact the basal forebrain: (1) BFCN nuclear size and neuronal numbers are decreased in aged rats (Altavista et al., 1990); (2) TrkA receptor and downstream NGF signaling protein levels are decreased in aged rat BFCNs (Williams et al., 2006, 2007; Parikh et al., 2013); (3) aging reduces the length of cholinergic fibers projecting to the hippocampus from the basal forebrain (Ypsilanti et al., 2008); and (4) an impaired retrograde axonal transport is observed in aged rat BFCNs (Cooper et al., 1994; De Lacalle et al., 1996; Bearer et al., 2018; Shekari and Fahnestock, 2019). In conclusion, the results of the present study, in agreement with previous ones, indicate that proper, balanced NGF/TrkA signaling is required for the correct functioning of cholinergic BF function. Therefore, NGF/TrkA system and its downstream pathway components could be suitable targets for the development of treatments for normal aging and diseases implicating the cholinergic system.

## Data availability statement

The raw data supporting the conclusions of this article will be made available by the authors, without undue reservation.

## Ethics statement

The animal study was approved by Comité de Bioética de la Universidad de Salamanca. The study was conducted in accordance with the local legislation and institutional requirements.

## Author contributions

LC-E: Conceptualization, Investigation, Methodology, Writing – original draft, Writing – review & editing, Data curation. SL: Investigation, Writing – review & editing. CV-G: Investigation, Writing – review & editing. RD: Resources, Writing – review & editing. JCA: Conceptualization, Funding acquisition, Investigation, Methodology, Writing – original draft, Writing – review & editing.
