# Supplementary material for: Enhanced TrkA signaling impairs basal forebrain-dependent behavior
Source: Front Mol Neurosci. 2023 Sep 22;16:1266983. doi: 10.3389/fnmol.2023.1266983 (PMC10556247; doi:10.3389/fnmol.2023.1266983)
Supplement: Supplementary file 1 [file Data_Sheet_1.PDF]

Supplementary Material

Enhanced TrkA signaling results in impaired basal forebrain-dependent behavior

Laura Calvo-Enrique[1,2*], Silvia Lisa[1,2], Cristina Vicente-García[1,2], Rubén Deogracias[1,2] & Juan Carlos Arévalo[1,2*]

[1]Instituto de Neurociencias de Castilla y León (INCyL), Department of Cell Biology and Pathology, Universidad de Salamanca, Spain.
[2]Institute of Biomedical Research of Salamanca (IBSAL), Salamanca, Spain.

*** Correspondence:**
Corresponding Authors:
Laura Calvo-Enrique (lau@usal.es); Juan Carlos Arévalo (arevalojc@usal.es)

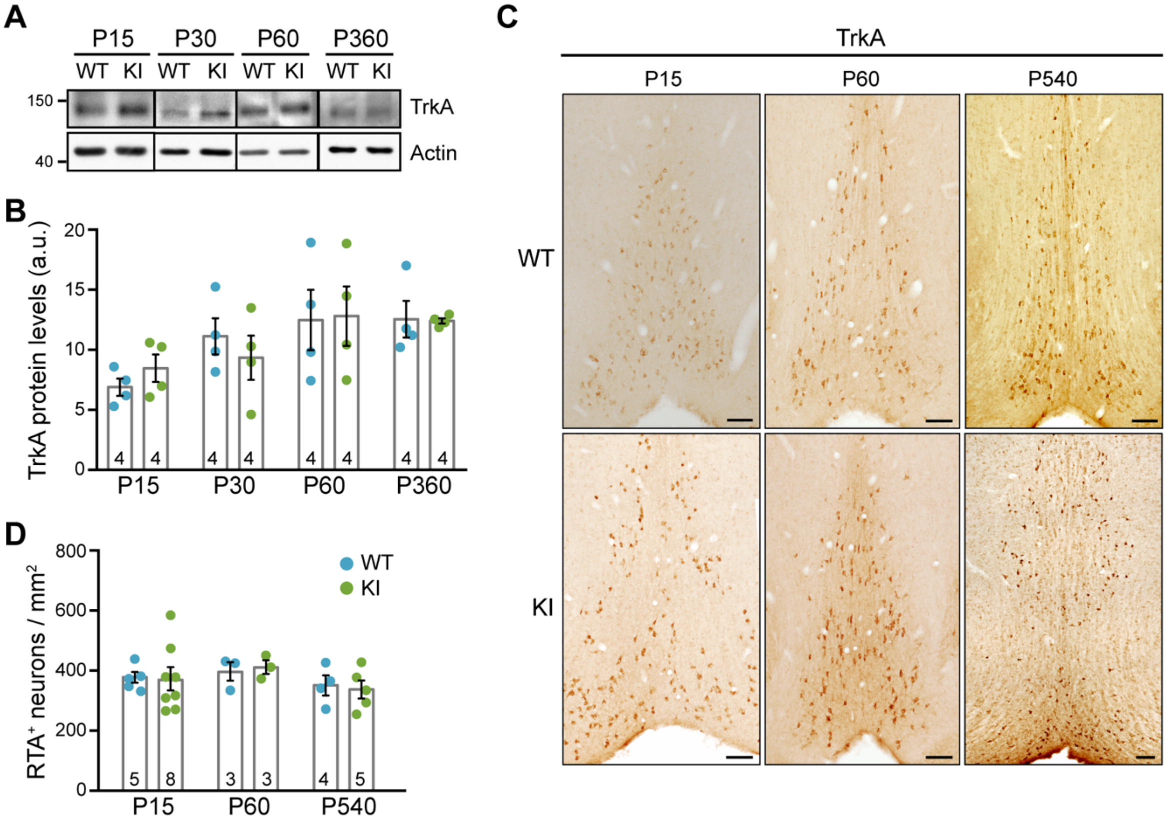

Supplementary Figure 1. TrkA protein levels nor TrkA-expressing neurons in the MS are altered in the TrkA KI mice. **A)** Western blot showing TrkA protein levels between P15 and P360 in WT and KI mice. Actin was used as loading control. **B**) Quantification of TrkA protein levels at different time points (*n*=4). **C**) Representative images of immunohistochemistry against TrkA in the MS of WT and KI mice at different ages. **D)** Quantification of TrkA-positive neurons per area at different ages in WT and KI mice (*n*=3-8 per age and genotype as indicated in inside bars).
